# NCK2 Is Significantly Associated with Opiates Addiction in African-Origin Men

**DOI:** 10.1155/2013/748979

**Published:** 2013-02-28

**Authors:** Zhifa Liu, Xiaobo Guo, Yuan Jiang, Heping Zhang

**Affiliations:** ^1^Department of Biostatistics, Yale University School of Public Health, New Haven, CT 06520, USA; ^2^Department of Statistical Science, School of Mathematics and Computational Science, Sun Yat-sen University, Guangzhou 510275, China; ^3^Department of Statistics, Oregon State University, Corvallis, OR 97331, USA

## Abstract

Substance dependence is a complex environmental and genetic disorder with significant social and medical concerns. Understanding the etiology of substance dependence is imperative to the development of effective treatment and prevention strategies. To this end, substantial effort has been made to identify genes underlying substance dependence, and in recent years, genome-wide association studies (GWASs) have led to discoveries of numerous genetic variants for complex diseases including substance dependence. Most of the GWAS discoveries were only based on single nucleotide polymorphisms (SNPs) and a single dichotomized outcome. By employing both SNP- and gene-based methods of analysis, we identified a strong (odds ratio = 13.87) and significant (*P* value = 1.33*E* − 11) association of an SNP in the *NCK2* gene on chromosome 2 with opiates addiction in African-origin men. Codependence analysis also identified a genome-wide significant association between *NCK2* and comorbidity of substance dependence (*P* value = 3.65*E* − 08) in African-origin men. Furthermore, we observed that the association between the *NCK2* gene (*P* value = 3.12*E* − 10) and opiates addiction reached the gene-based genome-wide significant level. In summary, our findings provided the first evidence for the involvement of *NCK2* in the susceptibility to opiates addiction and further revealed the racial and gender specificities of its impact.

## 1. Introduction

Substance dependence is believed to result from a combination of genetic and environmental factors. Since substance dependence is a chronic brain disease, with high relapse rates, it causes serious social, economic, and medical consequences [1–3]. The World Health Organization (WHO) and the United Nations Office on Drugs and Crime (UNODC) reported that opiates dependence is associated with a high risk of HIV infection when opiates are injected using contaminated injection equipment [4]. Paulozzi et al. in 2006 reported that the number of deaths which involved prescription opioid analgesics increased from 2,900 in 1999 to at least 7,500 in 2004, an increase of 160% in just 5 years [5]. All available evidence indicated that the increasing numbers of deaths are significantly correlated to the increasing use of prescription drugs, especially opioid painkillers, among people during the working years of life. While exposure to drugs is the prerequisite for addiction, the most important question is as follows: who will be addicted after the exposure? Genes are believed to be a major factor, although it is most likely that there are multiple genes as well as gene-environment interactions. For this reason, understanding the genetic mechanisms behind vulnerability to drug addiction is critical to improve the quality of overall health and life.

Linkage and genome-wide association studies (GWASs) have implicated many regions and genes for dependence on alcohol, tobacco, and opiates. GABRA2, CHRM2, ADH4, PKNOX2, GABRG3, TAS2R16, SNCA, OPRK1, and PDYN have all been associated with alcohol dependence with various degrees of replication [6–21]. Associations of other candidate alcohol dependence genes, such as KIAA0040, ALDH1A1, and MANBA [18, 20, 22–25], remain to be confirmed. Several groups reported CHRNA5, CHRNA3, CHRNB4, and CSMD1 to be associated with nicotine dependence [26–34]. Meanwhile, recent studies also reported that a group of genes, such as OPRM1 [35–37], OPRD1, OPRK1 [21, 38, 39], HTR1B [40], SLC6A4 [41], GABRG2 [42], and BDNF [43], to be associated or in linkage with opiates addiction.

Complex diseases may involve heterogeneous genetic effects in different ethnic and gender groups [7, 44–47]. Luo et al. [44] reported that African-origin smokers become dependent at a lower threshold (number of cigarettes per day) than European-origin smokers. Hartel et al. [46] found that men are more vulnerable to addiction when compared to women. In addition, Chen et al. [7] revealed that *PKNOX2* is associated with drug addiction in European-origin women. These examples underscore the necessity to consider demographic or even other covariates in genetic association studies.

Many of the reported genetic variants have been identified through single SNP association tests. Despite many of the successes, a single SNP tends to have a small effect, and the single SNP-based association tests require a very stringent significance level, which is likely a key factor to the so-called “missing heritability” problem [48, 49]. To overcome some of these limitations, gene-based analysis [50–52] has emerged to jointly analyze the SNPs within genes. Gene-based methods are less affected by the heterogeneity of a single locus; hence the results may be more robust across populations [53], which increases the likelihood of replication. Hence, we performed both single SNP-based and gene-based association analyses for the data from the Study of Addiction: Genetics and Environment (SAGE) [6] which includes well-characterized phenotypic data on substance dependence including addiction to nicotine, alcohol, marijuana, cocaine, opiates, and other drugs. In our analysis, we find a genome-wide significant association of *NCK2* gene on chromosome 2 with opiates dependence in African-origin men at both the SNP and gene levels. *NCK2* is a member of NCK family of adaptor proteins, which is associated with tyrosine-phosphorylated growth factor receptors of their cellular substrates [54]. However, to the best of our knowledge, *NCK2* has not been reported to be associated with any drug addiction outcomes in humans. 

## 2. Materials and Methods

Phenotypes for multisubstance dependency and genome-wide SNP data from SAGE [6] were downloaded from dbGaP (http://www.ncbi.nlm.nih.gov/gap). SAGE is a large case-control association study which investigates the genetic variants for drug addiction. The samples were collected from three large-scale genome-wide association studies: Collaborative Study on the Genetic of Alcoholism (COGA), the Family Study of Cocaine Dependence (FSCD), and the Collaborative Genetic Study of Nicotine Dependence (COGEND) [16, 44, 55, 56]. The original data set contains 4,121 subjects with six categories of substance dependence data: addiction to alcohol, cocaine, marijuana, nicotine, opiates, and other drugs. Lifetime dependence on these six substances is diagnosed by the Diagnostic and Statistical Manual of Mental Disorders, Fourth Edition (DSM-IV). The genotyping was performed by the illumina Human 1 M platform. In this study, we followed a quality control/quality assurance process similar to previous analyses [7, 57]. Individuals with call rates <90% and SNPs with minor allele frequency MAF <1% were excluded from the analysis. The *P* value for the Hardy-Weinberg equilibrium was set up by >0.0001. These steps reduced the level of noise in genotypes and increased the efficiency of analysis. There are 60 duplicate genotype samples and 9 individuals with ethnic backgrounds other than African origin or European origin. All of those individuals were removed from the subject list. Finally, there were a total of 3,627 unrelated samples with 859,185 autosomal SNPs for our final analysis. To alleviate the confounding by population substructure, we stratified the sample by race and sex. Finally, there are four sub-samples: 1,393 European-origin women, 1,131 European-origin men, 568 African-origin women and 535 African-origin men. The distribution of subjects diagnosed with lifetime dependence on substances in each of the six categories: nicotine, alcohol, marijuana, cocaine, opiates, or other drugs are presented in Table 1. 

## 3. Methods

**Figure 1:**
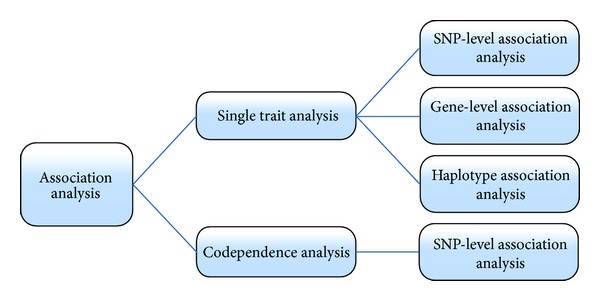
The pipeline of the association analysis.

Figure 1 displays the flow chart of our analytic strategy, and the details of the association analysis methods are described later. 

### 3.1. Statistical Analysis for Single Trait

The SNP-based association is performed by the standard allelic test and logistic regression to obtain the *P* values for individual SNPs, and PLINK software (version 1.07) was used for analysis [58]. Meanwhile, a list of SNP pairs in linkage disequilibrium (LD) (*r* ^2^ > 0.2) is calculated for the gene-based association test. 

For the gene-based analysis, we used the open-source tool: Knowledge-Based Mining System for Genome-Wide Genetic Studies (KGG, version 2.0)—based on the SNP association test results and LD files produced by PLINK. The procedure was performed as the following. We first calculate the effective number *m*_*e*_ of independent *P* value among *m* SNPs within a gene. Then, we sort the SNPs and calculate the effective number *m*_*e*(*j*)_ of independent *P*-values among the top *j* significant SNPs. Finally, the modified Simes test [51] was employed to obtain a gene-based *P* value as follows,

(1) $$\begin{array}{c} P_{G}=\mathrm{Min}\left(\frac{m_{e}P_{\left(j\right)}}{m_{e(j)}}\right), \end{array}$$

where *P*_(*j*)_ is the *j*th most significant among the *m* SNPs within a gene. We refer the interested readers to [51] for details. 

In the gene-based method, SNPs within 20 kilo bases (kb) 5′ upstream and 10 kilo bases (kb) 3′ downstream of a gene's coding regions [59] were assigned to the gene. In addition, we included other SNPs if they are in strong LD (*r* ^2^ > 0.9) with the initially mapped SNPs within the gene [60]. 

Since there are about 20,000 protein coding genes in human genome, we used 0.05/20, 000 = 2.5 × 10^−6^ as the genome-wide significance threshold for the gene-based association test. In contrast, we used 5.0 × 10^−8^ as the genome-wide significance threshold for the SNP-based association test [61]. 

### 3.2. Codependence Association Analysis

Although logistic regression is commonly used to study a binary outcome, it is not suitable to evaluate comorbidity involving multiple outcomes. We use a nonparametric association test based on Kendall's tau [62] to study the comorbidity. The Kendall's tau-based association test proceeds as follows.

Suppose that we observe a *p*-dimensional vector of traits *Y*_*i*_ = (*Y*_*i*_^(1)^,…, *Y*_*i*_^(*p*)^)^*T*^, genotype *G*_*i*_, and a *q*-dimensional vector of covariates *Z*_*i*_ = (*Z*_*i*_^(1)^,…, *Z*_*i*_^(*q*)^)^*T*^ for the *i*th subject in a population-based study with *n* subjects, and {(*Y*_*i*_, *G*_*i*_, *Z*_*i*_) : *i* = 1,…, *n*} are independent samples. For subjects *i* and *j*, let *Y*_*i*_ and *Y*_*j*_ be their vectors of traits, respectively, and analogously, *G*_*i*_ and *G*_*j*_ and *Z*_*i*_ and *Z*_*j*_ are their genotypes and covariates. Generalized from Kendall's tau, a *U* statistic is defined to measure the association between *Y* and *G* as follows:

(2) $$\begin{array}{c} U={\left(\begin{array}{c} n\\ 2 \end{array}\right)}^{-1}\sum_{i<j}\left(Y_{i}-Y_{j}\right)\left(G_{i}-G_{j}\right). \end{array}$$

Without considering the covariates and conditioning on all phenotypes, *U* follows an asymptotically normal distribution in the absence of association [63]. To accommodate covariates, a weighted *U* statistic has been developed [64, 65]. We refer to Jiang and Zhang [64] for a detailed description of the method. For the purpose of comparison, we present the results with and without considering age as the covariate. Recall that our analysis is stratified by ethnicity and gender.

## 4. Results

### 4.1. Association Analysis at SNP Level

Table 2 summarizes the top four significant SNPs (with *P* < 1.0 × 10^−4^) in gene *NCK2* on chromosome 2 (2q12) for opiates dependence in African-origin men. We identified a genome-wide significant SNP (rs2377339 with *P* = 1.33 × 10^−11^) for the opiates dependence in African-origin men by the allelic test. Logistic regression also yielded strong evidence for the association between the SNP rs2377339 (*P* = 1.01 × 10^−7^) and opiates dependence although the *P*-value did not reach the genome-wide significance threshold. In addition, Table 2 presents the association results for the other five addictions with the four candidate SNPs. None of the four SNPs appeared significantly associated with the other five substance addictions. 

### 4.2. Association Analysis at Gene Level

The gene-based association results are displayed in the last two rows of Table 2. Specifically, we included 39 SNPs in NCK2. The *P* values from the gene-*NCK2*-based tests that were obtained through the standard allelic test and logistic regression are 3.12 × 10^−10^ and 2.70 × 10^−6^, respectively. The gene-based *P* value from the standard allelic test reached the genome-wide significance at gene level. The gene-based *P* value through logistic regression is very close to the gene-based genome-wide significance level. Therefore, both methods provided significant evidence that supports the association between the *NCK2* gene and opiates dependence in African-origin men. For the addiction of the other five substances in African-origin men, nicotine dependence had the most significant association with the *NCK2* gene (*P* = 9.56 × 10^−3^).

### 4.3. Haplotypes Analysis

**Figure 2:**
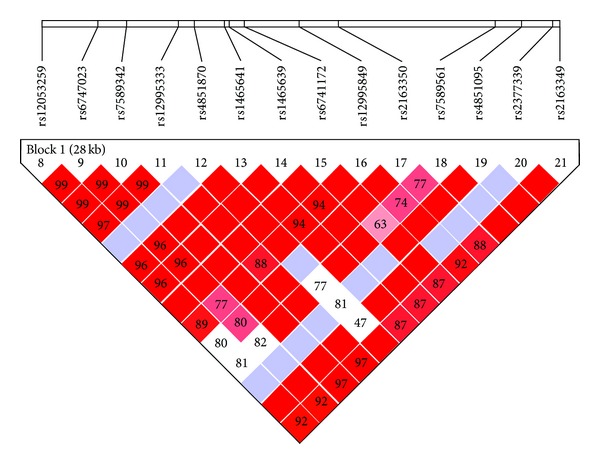
Linkage disequilibrium heat map near SNP rs2377339 on chromosome 2.

We also examined association of haplotypes with opiate addiction in *NCK2* region.  Figure 2 displays the linkage disequilibrium (LD) heat map of 14 SNPs in 28 kb region [66]. Haplotype “AGTTCAGATCTCGT” with probability 0.016 yielded a *P* value of 1.66 × 10^−11^. The genome-wide significant association between this haplotype and opiate addiction reduces the chance of a false discovery at the peak of a single SNP.

### 4.4. Contingency Table Analysis

 We further examined the relationship between SNP rs2377339 and the opiates dependence in African-origin men.  Table 3 depicts the allele frequencies of SNP rs2377339. The proportion of individuals having minor allele G is 21.43% in the case group and 1.63% in the control group. The odds ratio of SNP rs2377339 is 13.87, indicating that those who have the risk allele (G) for rs2377339 are at a significantly increased risk of being diagnosed with opiates dependence.

### 4.5. Stratification Analysis

Furthermore, in Table 4, we investigated the racial specificity and sex difference in the association between SNP rs2377339 and opiates dependence. This scrutiny required us to include all racial and gender groups. We observed that the MAF and *P* values vary between different races and genders. The association between rs2377339 and opiates dependence becomes less significant in the overall cohort, after we adjusted race and gender in logistic regression.

### 4.6. Codependence Association Analysis

In Table 5, we also presented the association results for *NCK2* and comorbidity of substance dependence. The most significant signal in *NCK2* was observed for SNP rs2377339 in men of African-origin with *P* = 3.65 × 10^−8^ in adjusted association test and *P* = 2.03 × 10^−8^ in unadjusted association test. *P* values of SNPs in *NCK2* for other ethnicity by gender groups were far from the genome-wide significance level and, hence, are omitted here.

## 5. Discussion

We found a genome-wide significant association between SNP rs2377339 and opiates dependence in African-origin men. The *NCK2* gene that contains SNP rs2377339 also achieved the genome-wide significance for opiates dependence at the gene level. For the addiction of the other five substances, nicotine dependence had the most significant association but not significant at the genome-wide level. 

*NCK2*, a member of NCK family of adaptor proteins, is reported to be associated with tyrosine-phosphorylated growth factor receptors of their cellular substrate [54]. The association between *NCK2* and nicotine dependence has been suggested in humans [67, 68]. Our finding coupled with those human studies enhances the plausibility of a causality relationship between *NCK2* and drug addiction. 

Importantly, about one-fifth of opiates addiction subjects in the African-origin men carried minor allele G of SNP rs2377339, which is more than 10-fold of the frequency in the nonopiates dependence group. This suggested that the minor allele G in SNP rs2377339 potentially elevates the risk for opiates dependence in African-origin men. We acknowledge that our analysis included only 44 African-origin men with opiates dependence. Therefore, it is important and necessary to validate our finding through independent and larger cohort studies. Specifically, there are two possible strategies to validate our finding. The direct approach is to replicate the association between SNP rs2377339 and opiates dependence in a larger cohort. An indirect approach is to evaluate whether SNP rs2377339 is associated with any substance dependence (opiates, alcohol, marijuana, etc.) as presented in Table 2. 

A distinction of our analysis is to consider simultaneously multiple substance addictions rather than a single substance. This approach, which is a realistic depiction of substance dependence, confirmed that a novel susceptibility gene, *NCK2* is significantly associated with substance dependence in African-origin men.

This study has several limitations. First, we stratified by ethnicity and sex, which reduced sample sizes and affected the power of our analysis. Nonetheless, the significant associations revealed in African-origin men are consistent with the notion that men may be socially more prone to environmental influences that promote substance use and thus more vulnerable to addiction [46]. Second, for SNP rs2377339, we observed heterogeneous genetic effects, suggesting interactions between race, sex, and the gene, because the association is much weakened after adjusting for race and gender. Such interactions have been suggested in other addiction research [44, 45, 47]. Again, our result further supports the importance to examine interactions among genes, race, and sex in addiction.

## Figures and Tables

**Table 1:** Descriptive statistics of the key variables in the SAGE dataset stratified by sex and race.

	Black men	White men	Black women	White women	Overall
*n*	535	1131	568	1393	3627
Age (SD) yr.	40.9 (8.2)	38.7 (10.3)	39.7 (6.7)	38.2 (9.1)	39 (9.1)
Alcohol (%)	62.1	62.3	39.4	31.1	46.7
Cocaine (%)	46.4	27.3	36.3	12.5	25.8
Marijuana (%)	25.4	25.2	13.7	8.7	17.1
Nicotine (%)	47.5	46.7	47.7	41.1	44.8
Opiates (%)	8.2	9.9	6.2	4.8	7.1
Other drugs (%)	11.4	18	6.5	9.4	11.9
No drug (%)	27.1	31	38.9	50.1	39

**Table 2:** Association of the most significant SNPs in the NCK2 gene with the six substances dependence in African-origin sen.

Gene	SNP	Method	Alcohol	Cocaine	Marijuana	Nicotine	Opiates	Others
NCK2	rs2377339	Logistic regression	2.46*E* − 2	5.09*E* − 2	4.48*E* − 2	7.01*E* − 2	1.10**E** − 7	3.84*E* − 3
		Standard allelic test	6.03*E* − 3	4.34*E* − 2	3.89*E* − 2	6.25*E* − 2	1.33**E** − 11	1.78*E* − 3
	rs7589342	Logistic regression	2.84*E* − 1	8.79*E* − 1	9.35*E* − 1	8.60*E* − 1	1.45*E* − 4	4.26*E* − 1
		Standard allelic test	2.81*E* − 1	8.78*E* − 1	9.34*E* − 1	8.59*E* − 1	5.39*E* − 5	4.22*E* − 1
	rs12995333	Logistic regression	1.71*E* − 1	7.51*E* − 1	7.16*E* − 1	9.86*E* − 1	1.89*E* − 4	4.69*E* − 1
		Standard allelic test	1.68*E* − 1	7.51*E* − 1	7.15*E* − 1	9.86*E* − 1	7.82*E* − 5	4.67*E* − 1
	rs12053259	Logistic regression	1.39*E* − 1	9.43*E* − 1	7.42*E* − 1	9.01*E* − 1	2.31*E* − 4	4.86*E* − 1
		Standard allelic test	1.33*E* − 1	9.42*E* − 1	7.39*E* − 1	9*E* − 1	8.67*E* − 5	4.80*E* − 1
NCK2	—	KGG-logistic	1.83*E* − 1	8.15*E* − 1	5.92*E* − 1	9.56*E* − 3	2.70**E** − 6	9.45*E* − 2
NCK2	—	KGG- Standard allelic test	1.41*E* − 1	8.16*E* − 1	4.83*E* − 1	8.71*E* − 3	3.12**E** − 10	4.17*E* − 2

**Table 3:** Allele counts of rs2377339 in cases (opiates dependence) and controls (nonopiates dependence) in African-origin men.

	Genotype AA	Genotype AG	Genotype GG	Total
Case	35	9	0	44
Control	483	8	0	491

**Table 4:** Association between SNP rs2377339 and opiates dependence by race and sex.

	MAF	*P* value	OR
African-origin men	1.59%	1.33*E* − 11	13.87
African-origin women	1.14%	1.64*E* − 1	2.82
European-origin men	6.77%	8.13*E* − 2	0.55
European-origin women	6.64%	6.95*E* − 1	1.14
Combined*	5.04%	4.37*E* − 1	1.17

*Logistic regression is used to adjust for sex and race.

**Table 5:** Association of most significant SNPs in NCK2 with codependence of six individual substances dependence outcomes (*P* value).

Gene	SNP	position	*P* value for unadjusted	*P* value for adjusted
NCK2	rs2377339	105823723	3.65**E** − 8	2.03**E** − 8
	rs6747023	105798288	5.43*E* − 3	2.58*E* − 2
	rs7589342	105799910	3.13*E* − 3	1.68*E* − 2
	rs12995333	105802798	3.81*E* − 3	1.83*E* − 2
